# Cystic Fibrosis Transmembrane Conductance Regulator (CFTR) Dysfunction in Human Diseases: Molecular Mechanisms and Pathophysiological Implications

**DOI:** 10.3390/cells15111034

**Published:** 2026-06-04

**Authors:** Md. Sohanur Rahman, Mohammed Daira

**Affiliations:** Department of Pathology, School of Medicine, University of Pittsburgh, Pittsburgh, PA 15213, USA; mhd26@pitt.edu

**Keywords:** CFTR, gene, human diseases, respiratory system

## Abstract

Cystic Fibrosis Transmembrane Conductance Regulator (CFTR) dysfunction is increasingly recognized as a key contributor to a broad spectrum of human diseases beyond classical cystic fibrosis (CF). CFTR is a cAMP-regulated chloride and bicarbonate ion channel expressed in both epithelial and non-epithelial tissues, where it regulates ion homeostasis, mucosal hydration, and cellular signaling. Both inherited CFTR mutations and acquired dysfunction resulting from environmental or inflammatory factors can disrupt these physiological processes and drive disease progression. Current evidence linking CFTR dysregulation to respiratory diseases, such as cystic fibrosis, chronic obstructive pulmonary disease (COPD), asthma, and HIV-associated airway disease, as well as cardiovascular, renal, neurological diseases, and cancer, is comprehensively discussed. Mechanistically, impaired CFTR function promotes oxidative stress, chronic inflammation, epithelial barrier dysfunction, altered mucociliary clearance, and dysregulation of signaling pathways, including NF-κB, TGF-β, PI3K/Akt, MAPK, and Wnt/β-catenin. In the context of HIV infection and cigarette smoke exposure, CFTR suppression is mediated in part by TGF-β signaling and miRNA-dependent mechanisms, resulting in compromised airway defense and increased susceptibility to pulmonary complications. Recent studies further demonstrate that CFTR dysregulation alters the expression of genes involved in fibrosis, inflammation, angiogenesis, and epithelial–mesenchymal transition (EMT). Notably, CFTR may act as either a tumor suppressor or a context-dependent oncogene, depending on tissue type and signaling milieu, highlighting its complex role in cancer biology. Advances in CFTR-targeted therapies, including potentiators, correctors, gene therapy, and combination approaches, have markedly improved outcomes in CF and may offer therapeutic potential for diseases associated with acquired CFTR dysfunction. We summarize the systemic consequences of CFTR dysregulation and the need for further mechanistic and translational research to clarify its role across diverse human diseases.

## 1. Introduction

**Figure 1:**
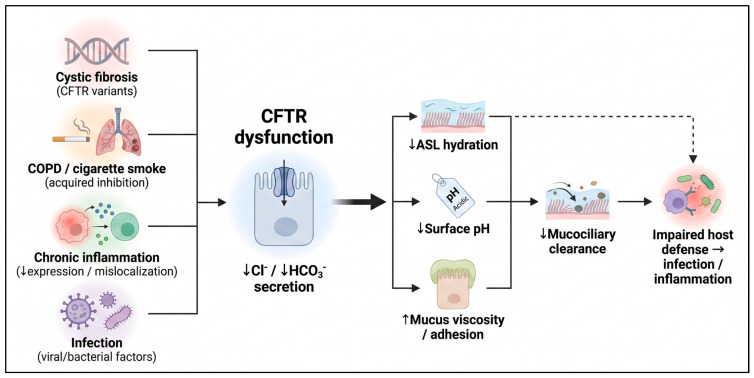
Mechanisms and consequences of CFTR dysfunction in inherited and acquired airway diseases. CFTR dysfunction results from either inherited CFTR mutations, as seen in cystic fibrosis, or from acquired suppression of CFTR activity due to cigarette smoke exposure, COPD, chronic inflammation, and viral or bacterial infections [30]. These factors diminish CFTR-mediated chloride (Cl^−^) and bicarbonate (HCO_3_^−^) secretion across epithelial surfaces, resulting in impaired airway surface liquid (ASL) hydration, decreased airway surface pH, and increased mucus viscosity and adhesion. These abnormalities collectively disrupt mucociliary clearance, compromise host defense mechanisms, and facilitate persistent infection and chronic inflammation [31]. Solid arrows in the figure denote mechanisms supported by direct experimental evidence, while dashed arrows indicate indirect or hypothetical downstream interactions that contribute to disease progression. Comparable mechanisms have been well documented in cystic fibrosis and are increasingly recognized in acquired CFTR dysfunction associated with smoking-related lung disease and chronic inflammatory airway disorders.

CFTR is a cAMP-regulated chloride and bicarbonate ion channel that belongs to the ATP-binding cassette transporter superfamily and plays a critical role in maintaining epithelial ion and fluid homeostasis [1,2]. CFTR is highly expressed in epithelial tissues of the airways, pancreas, intestine, and other organs, where it regulates mucociliary clearance (MCC), airway surface liquid composition, and epithelial barrier integrity [1,3]. Beyond epithelial cells, emerging evidence suggests that CFTR may also be expressed in non-epithelial tissues, including immune cells, smooth muscle cells, renal tissue, and neurons, although the physiological significance of CFTR in many of these tissues remains incompletely understood [3,4]. In addition to functioning as an ion channel, CFTR modulates the activity of other membrane transport proteins, including epithelial sodium channels (ENaC), thereby contributing to broader regulation of epithelial fluid balance [5]. Consequently, impaired CFTR expression or function disrupts ion transport and fluid homeostasis, leading to mucus dehydration, defective MCC, chronic inflammation, and progressive tissue injury [6]. Inherited mutations in the CFTR gene cause CF, a multisystem genetic disorder characterized primarily by chronic respiratory disease, pancreatic dysfunction, gastrointestinal abnormalities, and reduced life expectancy [6]. CFTR mutations are commonly categorized into six major functional classes based on the underlying molecular defect, including impaired protein synthesis, abnormal folding and trafficking, defective channel gating, and reduced channel stability [6]. The most prevalent pathogenic variant, F508del, results in the deletion of phenylalanine at position 508, causing protein misfolding, endoplasmic reticulum retention, and proteasomal degradation [7,8,9]. Even partial loss of CFTR activity can impair MCC and increase susceptibility to airway inflammation and infection, contributing to chronic respiratory diseases such as CF, chronic bronchitis, and COPD (Figure 1) [10].

In contrast to inherited CFTR mutations in classical CF, acquired CFTR dysfunction occurs in the absence of CFTR gene mutations and is increasingly recognized in chronic inflammatory and environmental exposure-related diseases (Figure 1) [11]. Cigarette smoke (CS), HIV infection, oxidative stress, and inflammatory signaling pathways have all been shown to suppress CFTR expression and function through post-transcriptional and epigenetic mechanisms (Figure 1) [11,12]. CS contains thousands of toxic compounds, including cadmium (Cd), a heavy metal that induces the generation of reactive oxygen species (ROS) and mitochondrial dysfunction [13,14,15]. Because Cd accumulates in lung tissue with a prolonged biological half-life, chronic exposure may contribute to persistent CFTR suppression and airway injury [16]. Experimental studies further demonstrate that cigarette smoke reduces CFTR expression through oxidative stress and inflammatory signaling pathways that suppress CFTR transcription and channel activity [17]. Similarly, HIV infection has been associated with impaired epithelial differentiation and reduced CFTR biogenesis and function [18]. HIV Tat protein suppresses CFTR expression partly through activation of TGF-β signaling and miRNA-mediated regulatory pathways, including increased expression of miR-145-5p and miR-509, which repress CFTR expression in bronchial epithelial cells [16,19,20,21,22]. These mechanisms may impair MCC and increase susceptibility to chronic airway infection and pulmonary comorbidities, even in patients receiving antiretroviral therapy [13]. Collectively, these findings support the concept that acquired CFTR dysfunction contributes to the pathogenesis of chronic airway disease independently of inherited CFTR mutations. The strongest clinical and mechanistic evidence linking CFTR dysfunction to disease pathogenesis exists in classical CF and acquired respiratory disorders such as COPD and smoking-related airway disease [10,17]. However, growing evidence suggests that CFTR dysregulation may also influence the pathogenesis of several extrapulmonary disorders, including cancer, cardiovascular disease, kidney disease, and neurological disorders [23]. Importantly, the strength of evidence supporting these associations varies substantially across disease systems. In cancer biology, CFTR has frequently been proposed to function as a tumor suppressor, particularly in gastrointestinal malignancies such as colorectal cancer (CRC), where patients with CF exhibit an elevated lifetime cancer risk [24,25]. Nevertheless, emerging studies also suggest that the role of CFTR in tumorigenesis may be tissue-specific and context-dependent, with both tumor-suppressive and potentially pro-tumorigenic effects reported depending on the signaling environment and cancer type [26]. In cardiovascular, renal, and neurological disorders, current evidence is derived predominantly from experimental models, in vitro studies, and indirect mechanistic observations, while direct clinical evidence linking CFTR dysfunction to disease progression remains comparatively limited [27]. Therefore, although CFTR dysregulation may contribute to inflammatory signaling, oxidative stress, fibrosis, and altered cellular homeostasis in these disorders, many of these associations remain mechanistically suggestive rather than clinically established.

Recent advances in CFTR-targeted therapies, including potentiators and correctors, have significantly improved outcomes for patients with CF carrying specific CFTR mutations [28]. However, the therapeutic application of CFTR modulation outside classical CF remains largely investigational. Although preclinical studies suggest that restoration of CFTR activity may provide benefit in smoking-related airway disease, HIV-associated pulmonary dysfunction, and other chronic inflammatory conditions, direct clinical evidence supporting these approaches is still limited [29]. Therefore, careful distinction between established therapeutic applications in CF and exploratory strategies in acquired or extrapulmonary CFTR dysfunction is essential. This review summarizes current knowledge of CFTR’s role in inherited and acquired disease states, with particular emphasis on the molecular mechanisms by which CFTR dysregulation alters gene expression, inflammatory signaling, and tissue homeostasis across multiple organ systems.

## 2. HIV Infection and Acquired CFTR Dysfunction in Airway Disease

HIV infection primarily targets CD4^+^ T lymphocytes through interactions with the CD4 receptor and the CCR5 or CXCR4 coreceptors, leading to progressive immune dysfunction and increased susceptibility to opportunistic infections [32,33,34,35]. Beyond systemic immune suppression, increasing evidence suggests that HIV infection also alters airway epithelial biology and contributes to acquired CFTR dysfunction through inflammatory and regulatory signaling pathways [36,37,38]. Unlike cystic fibrosis, where inherited CFTR mutations directly impair chloride transport, CFTR abnormalities in HIV-associated airway disease are considered acquired and are supported predominantly by experimental and mechanistic studies rather than established clinical evidence. Among the pathways implicated in HIV-associated CFTR suppression, TGF-β signaling has emerged as a central regulatory mechanism [19,37,38,39]. Experimental studies have demonstrated that HIV infection and HIV-derived proteins, particularly Tat, can enhance TGF-β signaling in airway epithelial cells, leading to reduced CFTR mRNA expression, impaired CFTR biogenesis, and diminished chloride channel activity [38,40,41]. Mechanistically, TGF-β-mediated suppression of CFTR appears to involve transcriptional repression as well as post-transcriptional regulation through microRNAs that target CFTR transcripts [38,41,42]. In vitro studies further suggest that pharmacologic inhibition of TGF-β signaling may partially restore CFTR expression and epithelial ion transport, supporting a functional relationship between TGF-β activation and acquired CFTR dysfunction [38,43].

Acquired suppression of CFTR in HIV-associated airway disease may contribute to impaired mucociliary clearance, airway surface dehydration, and reduced innate defense mechanisms within the respiratory epithelium [43]. These abnormalities could increase susceptibility to chronic airway inflammation and secondary pulmonary infections, particularly in individuals with additional environmental risk factors such as cigarette smoke exposure. Importantly, smoking and HIV infection appear to converge on overlapping inflammatory pathways involving oxidative stress and TGF-β signaling, resulting in additive suppression of CFTR function in experimental airway models [15,43]. However, although these mechanistic observations provide biological plausibility for CFTR involvement in HIV-associated lung disease, direct clinical evidence linking acquired CFTR dysfunction to respiratory outcomes in HIV-positive patients remains limited [12]. Therefore, current evidence supports CFTR dysregulation as a potentially important contributor to HIV-associated airway pathology, but its precise clinical significance and therapeutic implications require further validation in translational and clinical studies.

## 3. Involvement of CFTR in HIV-Associated Co-Morbidities

**Figure 2:**
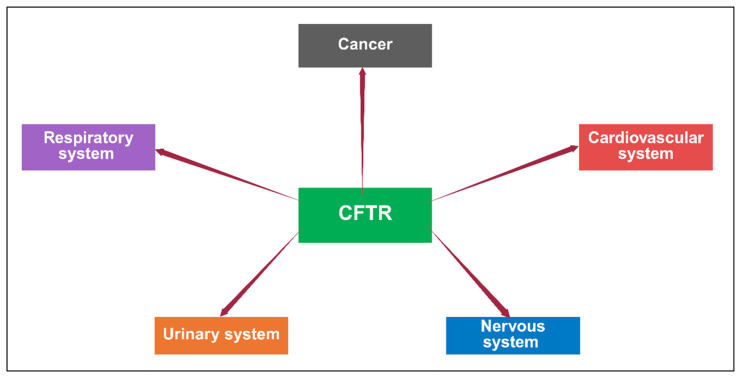
Schematic representation of the systemic consequences associated with CFTR dysfunction across multiple organ systems. Dysregulation of CFTR impairs epithelial ion transport, disrupts redox balance, and alters inflammatory signaling, contributing to pathophysiological changes in the respiratory, cardiovascular, urinary, and nervous systems. These alterations manifest as impaired mucociliary clearance, endothelial dysfunction, disturbed fluid homeostasis, and neuroinflammatory responses [46]. Chronic inflammation, oxidative stress, and disrupted cellular homeostasis together may create a pro-tumorigenic microenvironment, thereby associating CFTR dysfunction with an increased risk of cancer in multiple tissues [47].

Unlike CF, which is caused by inherited CFTR mutations, COPD and HIV-associated airway disease predominantly involve acquired CFTR dysfunction mediated by environmental exposure, oxidative stress, inflammatory cytokines, and epigenetic regulatory pathways [12,13,17,18]. Increasing evidence suggests that acquired CFTR suppression may contribute not only to chronic airway disease but also to broader systemic alterations affecting multiple organ systems, including cancer, cardiovascular, urinary, and nervous system disorders (Figure 2) [27,44,45].

## 4. Respiratory Diseases Due to Acquired CFTR Dysfunction

### 4.1. Impacts of Dysregulated CFTR in COPD

COPD is a prevalent respiratory disease characterized by persistent airflow limitation that commonly manifests as chronic bronchitis and/or emphysema [48]. In both phenotypes, mucus obstruction and small airway remodeling contribute substantially to impaired airflow and are frequently accompanied by comorbid conditions, including pulmonary hypertension, recurrent pneumonia, and asthma overlap syndromes [49,50]. Unlike CF, where CFTR dysfunction results from inherited pathogenic CFTR mutations, COPD is associated primarily with acquired CFTR suppression caused by environmental exposures, particularly cigarette smoke [30]. Evidence supporting acquired CFTR dysfunction in COPD is strongest in airway epithelial systems and chronic bronchitis phenotypes, where cigarette smoke exposure has consistently been shown to reduce CFTR expression and channel activity in vitro, in smoke-exposed animal models, and in observational studies involving smokers and patients with COPD [51,52,53]. These studies demonstrate that cigarette smoke impairs CFTR-mediated chloride and bicarbonate transport, leading to airway surface liquid dehydration and acidification [52,53]. Because bicarbonate secretion is required for appropriate mucin unfolding and maintenance of airway surface pH, impaired CFTR activity promotes mucus stasis, defective mucociliary clearance, and impaired innate host defense [54]. Experimental studies further suggest that this altered microenvironment compromises immune cell motility and phagocytosis, thereby facilitating chronic bacterial colonization and sustained airway inflammation [55,56,57]. Although these mechanistic observations are well supported by experimental airway models, the precise contribution of acquired CFTR dysfunction to long-term COPD progression in humans remains incompletely defined.

**Figure 3:**
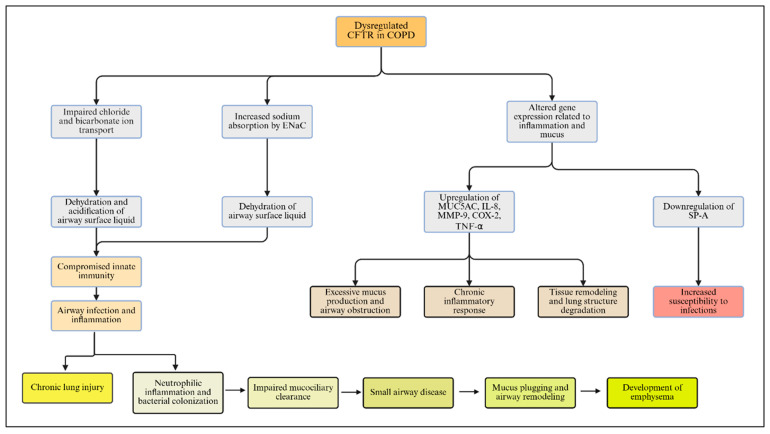
Mechanistic overview of COPD pathophysiology resulting from CFTR dysfunction. Decreased CFTR activity impairs chloride and bicarbonate transport and increases ENaC-mediated sodium absorption, leading to dehydration and acidification of the airway surface liquid [71]. These changes compromise mucociliary clearance and innate immunity, thereby increasing susceptibility to infection and neutrophilic inflammation [72]. Altered expressions of genes such as MUC5AC, IL-8, MMP-9, COX-2, and TNF-α, along with reduced SP-A, further promote mucus hypersecretion, inflammation, and tissue remodeling [73]. Collectively, these processes contribute to airway obstruction, small airway disease, and emphysema.

Airway remodeling in COPD also involves epithelial reprogramming and structural alterations that may further suppress CFTR expression (Figure 3). Single-cell transcriptomic studies have demonstrated the replacement of normal conducting airway epithelium by squamous epithelial populations with reduced CFTR expression during disease progression [58,59]. In this context, acquired CFTR dysfunction has been proposed to influence several inflammatory and mucus-associated signaling pathways; however, many of these molecular alterations overlap with broader inflammatory mechanisms characteristic of COPD and therefore cannot always be attributed specifically to CFTR suppression [1,30]. Among the best-characterized downstream effects is mucus hypersecretion mediated through EGFR and NF-κB activation. Experimental airway epithelial studies indicate that CFTR dysfunction promotes oxidative stress and airway dehydration, which activate EGFR and NF-κB signaling and, in turn, induce MUC5AC overexpression, thereby contributing to mucus accumulation, impaired mucociliary clearance, and progressive airway obstruction (Figure 3) [60,61,62]. Similarly, IL-8 is consistently elevated in COPD and contributes to neutrophilic inflammation and chronic tissue remodeling [63], although its upregulation is not unique to CFTR-dependent mechanisms. Additional experimental studies suggest that acquired CFTR dysfunction may activate NF-κB and MAPK signaling pathways, thereby increasing expression of TNF-α, COX-2, and MMP-9, molecules implicated in chronic inflammation, extracellular matrix degradation, and airway remodeling (Figure 3) [64,65,66,67]. Nevertheless, most evidence supporting these pathways derives from in vitro airway epithelial systems, cigarette smoke exposure models, or associative clinical observations rather than direct causal studies in human COPD tissue. Conversely, surfactant protein A (SP-A), which contributes to surfactant stability and pulmonary innate immunity, has been reported to be downregulated in association with CFTR dysfunction (Figure 3) [68]. Reduced SP-A expressions may impair surfactant homeostasis, gas exchange, and antimicrobial defense, thereby increasing susceptibility to recurrent infection and progressive lung injury [68,69,70]. Collectively, current evidence supports a mechanistic role for acquired CFTR dysfunction in mucus dehydration, impaired host defense, and inflammatory airway remodeling in COPD, particularly in chronic bronchitis phenotypes. However, many downstream inflammatory and remodeling-associated pathways remain incompletely validated as CFTR-specific mechanisms in human disease, and interpretation should consider the experimental context and varying strength of evidence across studies.

### 4.2. Asthma

CFTR is primarily recognized as a chloride and bicarbonate channel expressed in epithelial cells, where it regulates airway surface hydration and mucociliary clearance [74]. Inherited loss-of-function mutations in the CFTR gene cause CF, a monogenic disorder characterized by impaired ion transport, dehydrated mucus, chronic infection, and progressive airway inflammation [75,76]. In contrast, acquired CFTR dysfunction refers to secondary suppression or impairment of CFTR activity in individuals without classical CF-causing mutations and has been reported in chronic airway diseases such as asthma and COPD, particularly in the setting of cigarette smoke exposure and chronic inflammation [77]. Although heterozygous CFTR mutation carriers generally do not develop classical CF, several clinical and epidemiological studies suggest that some carriers may exhibit increased susceptibility to obstructive airway disease, including asthma; however, these associations remain variable across populations and are not yet considered clinically definitive [78]. Both inherited and acquired CFTR dysfunction can impair mucociliary clearance, promoting mucus retention, microbial colonization, and persistent inflammatory signaling within the airways [76,79]. Experimental studies using airway epithelial cells and animal models have demonstrated that reduced CFTR activity may enhance the production of Th2-associated cytokines, including IL-4 and IL-13, which contribute to eosinophilic inflammation, goblet cell hyperplasia, mucus hypersecretion, and airway hyperresponsiveness characteristic of asthma [80,81]. Among these mediators, IL-13 has been particularly implicated in epithelial remodeling by inducing mucus-associated genes, such as MUC5AC, and promoting goblet cell differentiation [82]. In addition, inflammatory signaling pathways involving EGFR activation further amplify epithelial responses to allergens and cytokines, thereby worsening mucus production and airway remodeling [83]. Several studies suggest that CFTR dysfunction may interact with profibrotic signaling pathways. Increased TGF-β expression has been observed in experimental models of reduced CFTR activity and in patients with CF airway disease, where it is associated with fibrosis, extracellular matrix deposition, and EMT [84,85]. Downstream mediators such as SMAD3 play central roles in transducing TGF-β signaling and have been linked to airway remodeling and collagen accumulation in asthma [86]. However, it is important to note that much of the evidence connecting CFTR dysfunction to TGF-β/SMAD3 signaling derives from mechanistic and associative studies rather than direct causal clinical investigations in asthma populations [87].

Chemokines, including CCL17, CXCL10, RANTES (CCL5), and IL-8, have also been reported to be elevated in inflammatory airway conditions associated with impaired CFTR function [88,89,90]. These mediators recruit eosinophils, neutrophils, and T lymphocytes into the airways, thereby sustaining chronic inflammation. Nevertheless, several of these inflammatory markers are not specific to CFTR dysfunction and may instead reflect broader airway inflammatory responses; therefore, caution is required when interpreting them as direct downstream consequences of CFTR dysregulation [91]. Similarly, modifier genes and polymorphic variants, including the CFTR M470V polymorphism, may influence CFTR biogenesis and phenotypic variability, potentially modifying airway reactivity and asthma susceptibility [92]. However, these genetic interactions remain incompletely understood and likely represent context-dependent modulators rather than independent disease-causing mechanisms. The current evidence supports a role for CFTR dysfunction in promoting mucus stasis, epithelial inflammation, and airway remodeling in obstructive lung disease. However, the strength of evidence varies substantially between classical CF, where causality is well established, and acquired CFTR dysfunction in asthma, where many mechanistic links remain experimental or associative rather than clinically validated. Future studies integrating human clinical data with mechanistic investigations will be necessary to clarify the precise contribution of CFTR dysregulation to asthma pathogenesis and disease heterogeneity. The consequences of the CFTR dysregulation are summarized in Table 1.

## 5. Emerging Systematic Diseases

### 5.1. Cancer

CFTR plays a complex, tissue-dependent role in cancer biology, functioning predominantly as a tumor suppressor in some epithelial malignancies while potentially exerting context-dependent pro-survival or oncogenic effects in others [99]. The tumor-suppressive role of CFTR is most consistently supported in gastrointestinal and respiratory cancers, particularly colorectal cancer and non-small cell lung cancer (NSCLC), where reduced CFTR expression, impaired channel activity, and promoter hypermethylation correlate with advanced disease stage, EMT, enhanced proliferation, and poor prognosis [47,100,101]. Experimental evidence from colorectal cancer cell lines, intestinal-specific Cftr-deficient murine models, NSCLC tissues, and human tumor specimens further demonstrate that CFTR deficiency promotes activation of the Wnt/β-catenin and inflammatory signaling pathways, which facilitate tumor progression [101,102,103]. Supporting this tumor-suppressive model, multiple studies have identified cancer-specific epigenetic silencing of CFTR through promoter hypermethylation in hepatocellular carcinoma, bladder cancer, prostate cancer, NSCLC, breast cancer, and head and neck cancers using human tumor tissues, methylation-specific PCR analyses, and cancer cell models [104,105,106,107,108,109]. These findings collectively suggest that CFTR loss may contribute to epithelial carcinogenesis by disrupting ion homeostasis, epithelial barrier integrity, and inflammatory regulation.

In contrast, evidence from glioma, ovarian cancer, cervical cancer, and certain gynecological malignancies suggests that CFTR may also exhibit context-dependent oncogenic properties [100,110]. Studies employing glioma cell lines, xenograft mouse models, glioblastoma patient tissues, and ovarian cancer cohorts have reported that elevated CFTR expression can promote Akt/Bcl2-mediated anti-apoptotic signaling, enhance tumor cell viability, and correlate with advanced FIGO stage and poor differentiation [110]. However, these proposed oncogenic roles remain comparatively preliminary because they are derived largely from in vitro systems, limited patient cohorts, and xenograft models rather than large mechanistic clinical investigations [99,110,111]. Consequently, current evidence favors a tissue-specific and pathway-dependent model in which CFTR function is shaped by the local inflammatory microenvironment, hormonal signaling context, epithelial lineage, and epigenetic landscape rather than by a universal oncogenic or tumor-suppressive mechanism [99,100].

Mechanistically, CFTR dysregulation has been linked to several interconnected pathways involved in tumor initiation and progression, including Wnt/β-catenin, NF-κB, PI3K/Akt, oxidative stress, angiogenesis, and EMT signaling [99,112,113,114]. Impaired CFTR function promotes chronic inflammation and oxidative stress, which can destabilize p53 signaling, impair apoptosis and DNA repair, and enhance tumor cell survival and genomic instability [113]. Additional studies using murine intestinal cancer models, CFTR-deficient mice, epithelial cell systems, endothelial assays, and human tumor tissues further demonstrate associations between CFTR dysfunction and increased expression of MUC1, VEGF-A, COX-2, and inflammatory mediators that may contribute to angiogenesis, epithelial remodeling, and tumor-promoting survival signaling [31,103,115,116]. Nevertheless, many of these mechanistic relationships remain linked, and direct causal interactions in human cancers have not yet been fully established [99,114]. Importantly, despite the transformative success of CFTR modulators in cystic fibrosis, there is currently no robust clinical evidence demonstrating that these therapies reduce cancer incidence or alter cancer progression in humans [117,118]. Thus, extrapolation of CFTR-targeted therapies to oncology remains speculative at present. Future studies integrating longitudinal patient cohorts, organoid systems, single-cell analyses, and tumor microenvironment modeling will be essential to define the causal and translational significance of CFTR dysfunction across different cancer types. Impaired CFTR functions directly or indirectly alter the expression of various genes and signaling pathways, as summarized in Table 2.

### 5.2. Cardiovascular Diseases

Although impaired CFTR expression is classically associated with CF and respiratory dysfunction, accumulating evidence suggests that CFTR dysregulation also contributes to cardiovascular pathology through both inherited and acquired mechanisms. Unlike respiratory manifestations, where CFTR dysfunction is clinically established, cardiovascular involvement remains an emerging area of investigation supported primarily by experimental and preclinical evidence [122]. Recent studies have identified CFTR expression in endothelial cells, vascular smooth muscle cells (VSMCs), and cardiomyocytes, suggesting broader roles in vascular and cardiac physiology beyond epithelial ion transport [123]. However, compared with CF lung disease, evidence linking CFTR dysfunction to cardiovascular pathology remains limited and derives mainly from in vitro studies, animal models, transcriptomic analyses, and associative clinical observations rather than direct clinical causation studies [124]. Experimental findings suggest that altered CFTR expression may influence chloride homeostasis, vascular tone, myocardial remodeling, and inflammatory signaling [77,125]. Acquired CFTR dysfunction induced by oxidative stress and chronic inflammation has also been proposed to contribute to vascular abnormalities outside classical CF [30]. Liu and colleagues demonstrated that reduced CFTR expression enhanced PDGF-BB-induced VSMC proliferation and migration in vitro and promoted carotid neointimal formation in a rat balloon injury model, potentially through activation of the SGK1 and MAPK pathways [126]. Similarly, Kolur et al. identified reduced CFTR expression in heart failure (HF) samples through bioinformatic analysis of the GSE141910 dataset and RT-qPCR validation, suggesting a possible association between CFTR dysregulation and HF pathogenesis [127]. In addition, Lidington and colleagues reported that experimental mouse models of HF and subarachnoid hemorrhage (SAH), including ΔF508-CFTR mutant and CFTR knockout mice, exhibited reduced cerebral arterial CFTR expression associated with impaired cerebrovascular reactivity, increased vasoconstriction, and diminished cerebral perfusion [128]. CFTR knockout mice have also demonstrated intrinsic cardiac abnormalities, including diastolic dysfunction, increased heart weight, and impaired stress-induced contractility [129].

Mechanistically, CFTR dysregulation promotes cardiovascular injury primarily through chronic inflammatory, oxidative, and profibrotic signaling pathways. CFTR dysregulation has been associated with altered expression of several inflammatory and vascular remodeling mediators, summarized in Table 3. Increased IL-6 and TNF-α expression may contribute to chronic inflammatory responses associated with cardiovascular dysfunction [130]. Another experimental studies further suggest that CFTR dysregulation promotes oxidative stress and endothelial inflammation through NF-κB, MAPK, PI3K/Akt, and HIF-1α signaling pathways, leading to increased expression of MMPs, VEGF, COX-2, and sphingosine-1-phosphate (S1P)-related mediators [131]. These pathways have been implicated in vascular remodeling, endothelial dysfunction, plaque instability, angiogenesis, cardiac fibrosis, and atherosclerosis. However, many of these associations remain indirect and mechanistically inferred from studies of inflammation or vascular biology rather than conclusively established as CFTR-specific pathogenic mechanisms in human cardiovascular disease [27]. Altered SP-A expression has also been linked to endothelial dysfunction and cardiovascular remodeling in experimental systems [132]. Overall, current evidence supports a potential role for CFTR in vascular homeostasis, inflammatory signaling, and cardiac remodeling. Nevertheless, direct clinical evidence establishing CFTR dysfunction as an independent driver of cardiovascular disease remains limited. Therefore, cardiovascular manifestations associated with CFTR dysregulation should presently be considered exploratory and mechanistically evolving rather than clinically established.

### 5.3. Kidney Diseases

CFTR is primarily recognized as a chloride channel expressed in epithelial cells that regulates chloride and bicarbonate transport, thereby maintaining fluid balance and electrolyte homeostasis [138]. The strongest evidence linking CFTR dysfunction to human disease comes from classical CF, in which pathogenic CFTR mutations disrupt ion transport and produce thick, viscous secretions that affect multiple organs, including the kidneys [139]. Renal complications in CF patients, such as electrolyte imbalance, nephrolithiasis, glomerulosclerosis, tubulointerstitial fibrosis, and chronic kidney disease (CKD), are increasingly recognized clinically, particularly as improved survival has revealed long-term systemic complications [140,141]. In the kidney, CFTR is expressed throughout the nephron, where it interacts with sodium and potassium transport pathways and contributes to epithelial homeostasis and tubular fluid regulation [142]. Beyond classical CF, acquired or secondary CFTR dysfunction has also been implicated in chronic inflammatory and fibrotic disorders, as demonstrated by experimental studies showing altered epithelial signaling, impaired barrier integrity, and dysregulated repair responses. However, the role of CFTR in non-CF kidney diseases remains largely exploratory, as most available evidence derives from experimental cell culture systems, animal models, and mechanistic studies rather than large-scale human validation studies [143].

Current experimental evidence suggests that reduced CFTR expression or activity may contribute to renal fibrosis by activating pro-inflammatory and profibrotic signaling pathways, particularly TGF-β/Smad and Wnt/β-catenin signaling. Sustained Wnt/β-catenin activation is now recognized as a central driver of CKD progression and renal fibrogenesis across multiple experimental models [144]. In CFTR-related kidney studies, CFTR downregulation has been shown to induce EMT, suppress E-cadherin, disrupt epithelial junction integrity, and enhance fibrotic gene expression in unilateral ureteral obstruction (UUO) mouse models, ΔF508 CFTR mutant mice, and renal tubular epithelial cell lines, including HK-2 and MDCK cells [143]. Mechanistically, CFTR appears to negatively regulate β-catenin signaling through interaction with Disheveled-2 (Dvl2), and loss of CFTR activity promotes β-catenin nuclear accumulation and transcription of fibrosis-associated genes such as MMP7, MMP2, COL1A2, and COL3A1 [143]. Experimental CFTR suppression has also been associated with enhanced NF-κB, HIF-1α, and TGF-β signaling, contributing to altered MUC1 regulation, increased VEGF expression, abnormal angiogenesis, inflammation, and maladaptive epithelial remodeling in cultured renal epithelial and endothelial cells, as well as murine models of chronic kidney injury [143,145,146,147,148,149,150]. Additional genes implicated in CFTR-associated renal pathology include INHBA and SLC26A9, whose dysregulation may further promote fibrosis and impaired ion transport [146,147,148]. Nevertheless, these proposed mechanisms are supported predominantly by preclinical data, and direct clinical evidence linking CFTR dysfunction to non-CF kidney disease progression in humans remains limited. Impaired CFTR function alters the expression of several genes and signaling pathways associated with renal pathology, as summarized in Table 4.

### 5.4. Nervous System Diseases

CFTR expression is widely distributed throughout the nervous system, where it contributes to chloride transport, neuronal excitability, neuroendocrine regulation, and maintenance of cellular homeostasis [154]. Early studies established that CFTR is expressed in multiple brain regions and participates in the regulation of mood, memory, respiration, motor activity, and energy balance [155,156,157]. In classical CF, neurological manifestations are not generally considered primary features of the disease; however, mounting experimental evidence suggests that CFTR dysfunction may indirectly influence nervous system development and neuroimmune regulation. Studies using CFTR-deficient pig models demonstrated abnormalities in Schwann cell function and peripheral nervous system development, supporting a role for CFTR in neural maturation and peripheral nerve integrity [158]. Nevertheless, direct clinical evidence linking CFTR mutations to primary neurological disease in humans remains limited, and most current knowledge derives from experimental systems, pharmacological CFTR inhibition, or indirect mechanistic observations. More recent investigations have shifted from classical CF phenotypes toward acquired or secondary CFTR dysfunction associated with neuroinflammatory processes. Experimental in vitro and in vivo studies demonstrated that reduced CFTR expression is associated with impaired hippocampal dendritic architecture, decreased spine density, enhanced microglial activation, and increased inflammatory cytokine production [159]. Similarly, lipopolysaccharide-induced suppression of CFTR expression in microglia reduces postsynaptic density protein-95 (PSD-95), whereas pharmacological correction of CFTR partially restores synaptic protein expression [160]. Restoration of CFTR activity in animal models also improved neurobehavioral abnormalities, including memory impairment and altered spine density following systemic injury [161]. Additional studies demonstrated that pharmacological modulation of CFTR attenuates vascular rhythmic disturbances and circadian dysregulation after subarachnoid hemorrhage, suggesting broader roles for CFTR in neurovascular homeostasis [162]. Collectively, these findings support the concept that acquired CFTR dysfunction may amplify neuroimmune and synaptic abnormalities beyond those observed in classical CF.

At the mechanistic level, CFTR dysregulation appears to converge on inflammatory and oxidative stress pathways that are commonly implicated in neurodegenerative disorders. Loss of CFTR activity activates NF-κB, MAPK, PI3K/Akt, and the NLRP3 inflammasome signaling pathways, thereby promoting the production of pro-inflammatory mediators, including IL-1β, IL-6, IL-8, and TNF-α [163,164]. These pathways are known to contribute to microglial activation, blood-brain barrier dysfunction, mitochondrial injury, and neuronal degeneration, mechanisms widely recognized in disorders such as Alzheimer’s disease, Parkinson’s disease, and multiple sclerosis [165]. However, current evidence does not establish CFTR dysfunction as a direct causal factor in these disorders; rather, CFTR-related signaling abnormalities may intersect with broader neuroinflammatory pathways involved in neurodegeneration. Consistent with this interpretation, dysregulated CFTR expression has been associated with altered expression of several inflammation- and stress-related genes, including S100B, CISD1, COX-2, MT-ND4, and BDNF (Table 5). Upregulation of S100B may amplify RAGE-mediated inflammatory signaling and oxidative stress, whereas altered CISD1 and MT-ND4 expression may contribute to mitochondrial dysfunction and impaired redox balance [166,167,168]. In parallel, increased COX-2 expression and reduced BDNF signaling may exacerbate neuronal injury, impair synaptic plasticity, and promote chronic neuroinflammation via NF-κB, MAPK, CREB, and PI3K/Akt signaling [116,169,170,171]. Together, these findings suggest that CFTR dysfunction may act primarily as a modifier of neuroimmune and mitochondrial signaling rather than as an independent driver of neurological disease.

## 6. Therapeutic Approaches Targeting CFTR

While this review discusses CFTR dysfunction in multiple human diseases, therapeutic development and clinical validation remain most advanced for classical CF caused by inherited CFTR mutations. Currently approved CFTR-targeted therapies, including potentiators and correctors, were specifically developed and clinically validated for CF patients carrying defined CFTR mutations. In contrast, extending these therapeutic strategies to acquired or secondary CFTR dysfunction observed in COPD, HIV-associated airway disease, cancer, cardiovascular disorders, kidney diseases, and neurological disorders remains largely investigational and is currently supported primarily by preclinical, mechanistic, or limited translational studies rather than established clinical evidence [178,179]. CF, caused by mutations in the CFTR gene, has driven the development of several mutation-targeted therapeutic strategies that substantially improve patient outcomes. Among these, CFTR modulators represent a major therapeutic breakthrough (Table 6). These agents are designed to restore or enhance CFTR protein function, thereby improving chloride and bicarbonate transport across epithelial membranes [178,179]. CFTR modulators are generally categorized into potentiators, correctors, amplifiers, stabilizers, and read-through agents [179]. Potentiators, such as ivacaftor, increase the channel-opening probability of mutant CFTR proteins and significantly improve chloride transport in patients carrying gating mutations such as G551D [180]. Correctors, including lumacaftor and tezacaftor, improve folding and trafficking of misfolded CFTR proteins to the cell surface and are particularly effective in patients carrying the common F508del mutation [181]. The triple-combination therapy elexacaftor/tezacaftor/ivacaftor has demonstrated substantial clinical benefits in CF patients with one or more F508del alleles, including marked improvements in lung function, reduced pulmonary exacerbations, and improved quality-of-life measures in phase III clinical trials [182,183]. Clinical studies have reported approximately 14–15% increases in percent predicted forced expiratory volume in 1 s (ppFEV1), along with significant reductions in sweat chloride concentrations, reflecting restoration of CFTR activity [182,183,184]. Importantly, these clinical outcomes have been validated specifically in patients with cystic fibrosis and disease-causing CFTR mutations rather than in other disorders associated with acquired CFTR dysfunction [185,186,187].

Beyond CFTR modulators, gene therapy has emerged as a promising strategy to correct the underlying genetic defect in CF (Table 6). These approaches seek to deliver functional CFTR copies into airway epithelial cells using viral vectors such as adenoviruses and lentiviruses, or non-viral delivery systems including lipid nanoparticles [188]. In addition, genome-editing technologies such as CRISPR/Cas9 have enabled precise correction of pathogenic CFTR mutations at the genomic level [6,189]. Early experimental and translational studies have shown encouraging results in laboratory and preclinical settings, suggesting the potential for durable therapeutic correction [190]. However, despite this progress, gene therapy approaches remain largely experimental, and their long-term efficacy and safety in clinical practice continue to require further validation [6,188,189,190].

Adjunct therapies are also increasingly used to complement CFTR-targeted treatment strategies and improve overall patient management in CF (Table 6). These therapies address disease mechanisms that may not be fully corrected by CFTR modulators alone [6]. Anti-inflammatory therapies have been investigated to reduce chronic neutrophilic airway inflammation, which substantially contributes to progressive lung injury in CF [191]. Agents such as dornase alfa have demonstrated beneficial effects in reducing inflammatory burden and neutrophil-associated metalloproteinase activity [191]. Inhaled antibiotics remain essential for controlling chronic pulmonary infections common in CF patients and have been shown to improve clinical outcomes when combined with CFTR modulators [192,193]. Nutritional support is also critically important because pancreatic insufficiency frequently occurs in CF, necessitating pancreatic enzyme replacement therapy and high-calorie nutritional supplementation to maintain adequate nutritional status [194]. Together, these multidisciplinary therapeutic strategies improve respiratory outcomes, nutritional health, and overall quality of life in CF patients [195]. Importantly, although acquired CFTR dysfunction has been implicated mechanistically in diseases such as COPD, smoking-related airway disease, HIV-associated pulmonary disorders, cancer, cardiovascular disease, kidney disease, and neurological disorders, therapeutic application of CFTR modulators or gene-correction strategies in these conditions remains experimental. Current evidence for these non-CF diseases derives predominantly from cell culture systems, animal models, and limited early translational studies, and robust clinical trials demonstrating efficacy comparable to that observed in cystic fibrosis are currently lacking. Therefore, therapeutic extrapolation from CF to other CFTR-associated diseases should be interpreted cautiously until supported by direct clinical evidence.

**Table 6:** Therapeutic strategies targeting CFTR dysfunction, including clinically established and investigational approaches.

Approach	Type	Description	Status	References
CFTR Modulators	Small molecules	Small molecules, including potentiators, correctors, and triple-combination modulators, are designed to improve CFTR folding, trafficking, and channel activity	Established in CF	[196,197,198]
Gene Therapy	Genetic modification	Experimental approaches aimed at restoring CFTR expression or function through viral or non-viral genetic delivery systems	Investigational	[199,200]
Antisense Oligonucleotides	Nucleic acid-based	Synthetic oligonucleotides designed to modify CFTR mRNA splicing, stability, or processing to restore protein function	Preclinical/Early clinical evaluation	[201,202]
Lipid-based Therapies	Delivery systems	Liposomes and lipid nanoparticles are used to enhance the intracellular delivery of CFTR-targeted therapeutics or nucleic acids	Investigational	[198,200]
Combination Therapies	Multi-agent strategies	Combined therapeutic approaches integrating multiple modulators or adjunct therapies to improve CFTR rescue and clinical response	Clinically evaluated/Expanding	[203,204]

## 7. Ongoing and Completed CFTR-Focused Clinical Trials

Ongoing and completed clinical trials targeting the CFTR have primarily focused on CF, where inherited CFTR mutations directly impair chloride transport and lead to progressive respiratory and systemic disease [55,161]. The strongest clinical evidence for CFTR-targeted therapies currently exists in CF, particularly through the development of small-molecule modulators that restore or enhance mutant CFTR function [55,205]. Among these, Ivacaftor represented a major therapeutic breakthrough for patients carrying gating mutations such as G551D, significantly improving lung function, nutritional status, and pulmonary exacerbation rates in randomized clinical trials [55,199]. Subsequently, combination therapies, including Lumacaftor/Ivacaftor (Orkambi) and Tezacaftor/Ivacaftor, expanded treatment options for patients with the F508del mutation, although clinical responses varied by genotype and disease severity [179,206]. More recently, next-generation correctors such as VX-659 and related triple-combination regimens have demonstrated enhanced efficacy in improving CFTR processing and clinical outcomes in patients with more complex mutation profiles [207,208]. Collectively, these advances illustrate the success of precision medicine approaches in CF, in which therapy is tailored to specific CFTR genotypes.

In parallel, investigational therapeutic strategies continue to explore approaches beyond currently approved modulators. Gene therapy and mRNA-based interventions aim to restore functional CFTR expression by delivering corrected genetic material to airway epithelial cells [208,209]. Although preclinical and early clinical studies have shown promise, major challenges remain in delivery efficiency, expression durability, vector safety, and effective targeting of lung tissues. Similarly, patient-derived organoid models and high-throughput screening platforms are increasingly being used to evaluate individualized drug responses and identify novel CFTR-correcting compounds, supporting the development of personalized therapeutic strategies [209,210,211]. Combination approaches integrating CFTR modulators with anti-inflammatory therapies are also under investigation to address the chronic inflammatory environment characteristic of CF lung disease [206,212]. Importantly, while acquired CFTR dysfunction has been implicated in disorders such as COPD, HIV-associated airway disease, cancer, cardiovascular disease, kidney disease, and neurological disorders, the therapeutic application of CFTR-targeted strategies in these conditions remains largely investigational [120,154,213]. In many of these disease contexts, current evidence is derived predominantly from in vitro studies, animal models, indirect mechanistic observations, or associative clinical findings rather than established clinical trials. Therefore, although modulation of acquired CFTR dysfunction represents a biologically plausible therapeutic avenue, its clinical relevance outside CF has not yet been definitively established. Future studies will require careful validation through mechanistic investigations, translational models, and controlled clinical trials to determine whether CFTR-targeted interventions can provide meaningful therapeutic benefit beyond classical cystic fibrosis. All major CFTR-targeted clinical trials, their clinical status, and FDA approval timelines are summarized in Table 7.

## 8. Limitations and Challenges in Current CFTR Research

Several limitations currently limit the interpretation of CFTR-related disease associations beyond classical cystic fibrosis. For instance, much of the available evidence derives from in vitro studies, animal models, transcriptomic analyses, or indirect mechanistic observations, rather than large prospective clinical studies [220]. In many non-CF diseases, it remains difficult to determine whether CFTR dysfunction acts as a primary pathogenic driver, a secondary consequence of chronic inflammation, or an adaptive response to tissue injury [31]. Moreover, substantial heterogeneity exists across tissue types, experimental systems, and patient populations [221]. In addition, many reported gene associations are context-dependent and may reflect generalized inflammatory signaling rather than direct CFTR-mediated regulation [116]. Finally, the long-term impact of CFTR-targeted therapies in acquired CFTR dysfunction remains unknown. Addressing these limitations will therefore require integrated mechanistic, translational, and longitudinal clinical studies.

## 9. Conclusions and Future Directions

CFTR dysfunction plays a critical role in the pathogenesis of a wide range of diseases beyond classical cystic fibrosis, such as chronic respiratory, cardiovascular, kidney, and neurological disorders, as well as multiple cancers. Both inherited mutations and acquired CFTR suppression from factors like cigarette smoke, chronic inflammation, HIV infection, and oxidative stress disrupt ion transport, mucociliary clearance, epithelial integrity, and inflammatory signaling. Recent advances in CFTR modulators, gene therapy, and combination treatments have improved outcomes in cystic fibrosis and offer a promising approach for other diseases involving acquired CFTR dysfunction. The complex interplay among genetic, epigenetic, environmental, and inflammatory factors also remains poorly understood. Developing more physiologically relevant models, such as organoids, humanized animal models, and single-cell multi-omics approaches, is essential to better reflect human pathology. Further investigation into the epigenetic regulation of CFTR, including DNA methylation, histone modifications, and miRNA-mediated suppression, may identify new therapeutic targets and biomarkers. In cancer research, studies should define the tissue-specific dual role of CFTR as both a tumor suppressor and a potential oncogene, and assess whether CFTR modulators influence cancer initiation, progression, or treatment response. Translational studies are needed to evaluate the effectiveness of CFTR-targeted therapies in COPD and HIV-associated co-morbidities.

## Figures and Tables

**Table 1:** CFTR-associated inflammatory and epithelial genes implicated in asthma pathophysiology.

Gene	Regulation	Evidence	CFTR Association	Description	Reference
CCL17	Up	Human clinical samples, in vitro	Indirect	Contribute to the recruitment of Th2 cells and promote the allergic inflammatory response	[90]
CXCL10	Up	In vitro, human asthma samples	Indirect	CXCL10 may typically respond to inflammatory stimuli in the unique context of CFTR dysfunction related to asthma	[93]
IL-13	Up	In vitro, human airway tissue studies	Direct	Up-regulates CFTR-mediated Cl-secretion and CFTR expression; implicated in asthma pathophysiology	[94]
SMAD3	Up	In vitro, in vivo	Indirect	Involved in epithelial responses; upregulated in asthma exacerbations	[95]
MUC5AC	Up	In vitro, human asthma tissue studies	Indirect	Key mucin associated with mucus hypersecretion in asthma	[96]
EGFR	Up	In vitro	Direct	Associated with signaling pathways linked to asthma and mucus secretion	[97]
RANTES	Up	In vitro and inflammatory studies	Indirect	Regulates inflammatory responses in the airway epithelium	[98]
IL-8	Up	In vitro, human inflammatory airway samples	Direct	A pro-inflammatory cytokine involved in asthmatic inflammation	[97]
IL-10	Down	Human asthma samples	Indirect	An anti-inflammatory cytokine that may influence airway inflammation	[90]

**Table 2:** Cancer-associated genes dysregulated in relation to CFTR dysfunction and their experimental evidence.

Gene	Regulation	Evidence	CFTR Association	Description	References
p53	Down	In vitro and limited human tumor correlation studies	Indirect/Moderate	Frequently downregulated in various cancers, contributing to unchecked cellular proliferation when CFTR is dysfunctional	[119]
E-cadherin	Down	In vitro, in vivo	Direct	Maintain cell adhesion; its downregulation due to CFTR dysfunction may enhance cancer invasion	[102]
MUC1	Up	In vitro and human cancer tissue	Indirect	Oncogenic mucin that is often overexpressed in cancers, associated with poor prognosis and aggressive forms of tumors	[120]
VEGF	Up	In vitro, in vivo	Indirect/Moderate	Promotes angiogenesis; increased expression can enhance tumor growth and metastasis due to CFTR dysfunction	[121]
COX-2	Up	In vitro, in vivo	Indirect	Involved in inflammation and known to be overexpressed in several cancers, contributing to tumor progression	[116]
NF-κB	Up	In vitro, in vivo	Direct	The NF-κB pathway is often activated with CFTR dysfunction, leading to inflammation and tumor survival mechanisms	[119]

**Table 3:** Dysregulated inflammatory and vascular genes linked to CFTR dysfunction in cardiovascular disease.

Gene	Regulation	Evidence	CFTR Association	Description	References
IL-6	Up	In vitro, in vivo	Indirect	A pro-inflammatory cytokine is often associated with inflammation and cardiovascular risk	[133]
TNF-α	Up	In vitro, in vivo, and limited human observational data	Indirect	A key player in inflammation, TNF-α is often found to be upregulated in CFTR-related cardiovascular complications	[134]
MMPs	Up	In vitro, in vivo	Indirect	Involved in extracellular matrix remodeling, their expression typically increases in response to CFTR dysfunction	[135]
VEGF	Up	In vitro, in vivo, and some human tissue studies	Indirect	Promotes angiogenesis, often found elevated in the context of CFTR dysfunction, contributing to cardiovascular pathology	[121,133]
COX-2	Up	In vitro, in vivo	Indirect	Involved in inflammation and pain, typically upregulated in cardiovascular diseases linked with CFTR dysfunction	[136,137]

**Table 4:** Dysregulated renal genes associated with CFTR dysfunction and kidney fibrosis.

Gene	Regulation	Evidence	CFTR Association	Description	References
β-catenin	Up/Down	In vitro, in vivo, and human tissue	Direct	Altered β-catenin activity exacerbates kidney fibrosis by disrupting epithelial integrity, promoting apoptosis, and impairing renal repair	[134]
MUC1	Up	In vitro, some human genetic kidney disease studies	Indirect	This gene is often overexpressed in renal disease, associated with CFTR dysfunction, and contributes to inflammatory processes	[151]
INHB-A	Up	Animal models and human CKD/fibrosis studies	Indirect	Often found elevated in kidney diseases associated with CFTR dysfunction, involved in fibrosis	[152]
E-cadherin	Down	In vitro and in vivo	Direct	A critical cell adhesion molecule whose reduced expression may promote kidney damage and fibrosis	[143]
SLC26A9	Down	In vitro and animal models	Direct	A sulfate transporter is implicated in fluid balance and ion transport, which is often affected by CFTR dysfunction	[147]
VEGF	Up	In vitro and animal models	Indirect/Moderate	Increased expression is associated with angiogenesis and kidney injury, and is associated with CFTR dysfunction	[153]

**Table 5:** CFTR-associated inflammatory, mitochondrial, and neurodevelopmental genes implicated in nervous system disorders.

Gene	Regulation	Evidence Type	CFTR Association	Description	References
IL-1, IL-6, IL-8	Up	In vitro and in vivo	Indirect	Pro-inflammatory cytokines are associated with neuroinflammation	[97,172]
TNF-α	Up	In vitro and in vivo	Indirect	It can exacerbate inflammation and neuronal damage in CF-related disorders	[163,173]
S100B	Up	In vitro	Indirect	A protein linked to astrocyte activation and neuroinflammation	[173]
MUC1	Up	In vitro	Indirect	Involved in inflammatory responses, and its upregulation is linked to CFTR dysfunction and inflammation in the nervous system	[174]
MT-ND4	Down	In vitro	Indirect	Mitochondrial gene whose expression is reduced in CF, impacting mitochondrial function and neuronal health	[168]
CISD1	Down	In vitro	Indirect	Mitochondrial protein involved in cellular energy; downregulated in cases of CFTR deficiency, affecting neuronal function	[175]
COX-2	Up	In vitro and in vivo	Indirect	Involved in inflammation and pain pathways; often elevated in response to CFTR dysregulation in the nervous system	[176]
β-catenin	Up	In vitro and in vivo	Direct/Mechanistic	Associated with Wnt signaling; its aberrant activation is linked to neurodevelopmental disorders and fibrosis	[177]
BDNF	Down	In vitro and in vivo	Indirect	Reduced levels can impact neuronal survival and synaptic plasticity, often observed in CFTR-related neurodegenerative conditions	[172]

**Table 7:** Clinically Established and Investigational CFTR-Targeted Therapeutic Trials in Cystic Fibrosis and Emerging CFTR Modulation Strategies.

Trial/Therapy	Description	Status	FDA Approval Year	References
Ivacaftor (Kalydeco) for G551D Mutation	Evaluated the safety and efficacy of CFTR potentiation in patients carrying gating mutations such as G551D	Completed	2012	[55,199,214]
Lumacaftor/Ivacaftor (Orkambi) in F508del Patients	Combination corrector-potentiator therapy evaluated in homozygous F508del CF patients	Completed	2015	[185,186,206,214]
Tezacaftor/Ivacaftor (Symdeko/Symkevi) Study	Evaluated improved tolerability and efficacy in patients with specific CFTR genotypes	Completed	2018	[187,212,215]
VX-659 Triple-Combination Study	Investigated next-generation triple-combination regimens with Ivacaftor in F508del patients	Completed	2019	[207,210,215,216,217,218]
Elexacaftor/Tezacaftor/Ivacaftor (Trikafta/Kaftrio)	Triple-combination CFTR modulator therapy targets patients with at least one F508del mutation	Completed	2019	[55,161,207,208]
CFTR Gene Therapy Trials	Investigational gene-delivery approaches designed to restore functional CFTR expression in airway epithelial cells	Ongoing /Mixed Results	–	[208,209,219]
Emerging CFTR Modulators and mRNA Therapies	Early-phase studies evaluating novel modulators, mRNA therapies, and personalized CFTR-targeted strategies	Early Phase/Under Investigation	–	[209,210,211,217]

## Data Availability

No new data were created or analyzed in this study.

## Abbreviations

The following abbreviations are used in this manuscript:

CFTR	Cystic Fibrosis Transmembrane Conductance Regulator
CS	Cigarette smoke
CF	Cystic fibrosis
TGF-β	Transforming growth factor-beta
CRC	Colorectal cancer
AIDS	Acquired immunodeficiency syndrome
COPD	Chronic obstructive pulmonary disease
ENaC	Epithelial sodium channel
MMP9	Matrix Metalloproteinase-9
COX-2	Cyclooxygenase-2
SP-A	Surfactant Protein A
MUC5AC	Mucin 5AC
RANTES	Regulated upon Activation, Normal T-cell Expressed and Secreted
EGFR	Epidermal Growth Factor Receptor
HCCs	Hepatocellular carcinoma
NSCLC	Non-small cell lung carcinoma
EMT	Epithelial-to-mesenchymal transition
HF	Heart failure
LV	Left ventricular
MMPs	Matrix metalloproteinases
S1P	Sphingosine-1-phosphate
VEGF	Vascular endothelial growth factor
INHBA	Inhibin Subunit Beta A
PDGF	Platelet-Derived Growth Factor
PSD-95	Post-synaptic density protein 95
BALF	Bronchoalveolar lavage fluid
BDNF	Brain-Derived Neurotrophic Factor
